# Sequential anaphylaxis and delayed rituximab-induced serum sickness with skin testing findings: a case report

**DOI:** 10.1186/s13223-026-01050-0

**Published:** 2026-07-05

**Authors:** Shiori Mori, Utako Okata-Karigane, Masaki Arai, Chiaki Takahashi, Umi Tahara, Ayano Fukushima-Nomura, Eri Matsuki, Katsunori Masaki, Takeya Adachi

**Affiliations:** 1 Present Address: Department of Dermatology, Keio University School of Medicine, 35 Shinanomachi, Shinjuku-ku, Tokyo, 160-8582 Japan; 2 Allergy Center, Keio University Hospital, Tokyo, Japan; 3 Department of Hematology, Keio University School of Medicine, Tokyo, Japan; 4 Division of Pulmonary Medicine, Keio University School of Medicine, Tokyo, Japan

**Keywords:** Rituximab-induced serum sickness, Type III hypersensitivity, Anaphylaxis, Skin testing, Delayed hypersensitivity

## Abstract

**Background:**

Rituximab can cause immediate and delayed immune reactions, including rituximab-induced serum sickness (RISS). However, the temporal and mechanistic relationship between these reactions is unclear. We report a case in which RISS developed several days after rituximab-induced anaphylaxis.

**Case presentation:**

A 70-year-old woman with stage I mucosa-associated lymphoid tissue lymphoma was initially treated with weekly rituximab monotherapy. Four days after the third infusion, she developed fever, nonpruritic erythema, and arthralgia, which resolved spontaneously. When rituximab was re-administered, she immediately developed symptoms consistent with anaphylaxis, prompting treatment discontinuation. One year later, rituximab was re-started due to slight disease progression. The infusion induced nasal congestion and hoarseness but was completed under hydrocortisone. Eight days later, she presented with fever, widespread pruritic plaques, vomiting, diarrhea, and hypotension. Laboratory testing revealed elevated inflammatory markers without evidence of bacterial infection. Human anti-chimeric antibody levels were markedly elevated (> 5000 ng/mL). Intradermal testing with rituximab induced both an immediate wheal and a delayed erythematous flare lasting several days, indicating coexisting immediate-type hypersensitivity and RISS.

**Conclusions:**

This case demonstrates that RISS may occur several days after the resolution of rituximab-induced anaphylaxis. When immediate reactions occur after rituximab administration, patient education to ensure prompt reporting of delayed reactions is essential. Skin testing supported the identification of sequential immediate and delayed rituximab hypersensitivity in this case.

## Background

Rituximab is a chimeric IgG monoclonal antibody composed of murine and human components and directed against the CD20 antigen expressed on normal and malignant B cells. It is effective for the treatment of B-cell malignancies and autoimmune diseases [1]. Despite its efficacy and safety profile, adverse events, including immune-related complications, can lead to discontinuation. Rituximab-related hypersensitivity reactions encompass a broad spectrum, including type I, III, and IV hypersensitivity reactions, as well as infusion-related, cytokine-release, and mixed reactions [2]. Among these, immediate reactions specifically include cytokine-mediated reactions and both IgE- and non-IgE-mediated anaphylaxis. Rituximab-induced serum sickness (RISS) is an immune-related complication of rituximab, representing a type III hypersensitivity reaction [2]. RISS is characterized by fever, rash, and arthralgia, typically developing approximately 1 week after rituximab administration [3], more frequently in patients with underlying rheumatologic diseases than in those with hematological malignancies [4]. Diagnosis can be challenging owing to the lack of standardized diagnostic criteria. Human anti-chimeric antibodies (HACA), directed against the chimeric region of rituximab, are detected in approximately 60% of cases [3]. The clinical significance of HACA development remains unclear; however, it has been associated with an increased risk of infusion reactions and reduced therapeutic efficacy [5]. This complexity increases when multiple immune-related reactions occur concurrently. We herein report a case of RISS that developed several days after rituximab-induced anaphylaxis.

## Case presentation

A 70-year-old woman started rituximab monotherapy for stage I mucosa-associated lymphoid tissue lymphoma. The first three weekly doses of rituximab (375 mg/m^2^) were administered without any significant adverse events at a previous institution; however, 4 days after the third dose, the patient developed fever, nonpruritic flat erythema, and arthralgia. She visited a local clinic, but because these symptoms improved spontaneously within 2 days, no further infection workup was performed. Rituximab was re-administered 2 weeks after the third dose, following symptom resolution. 30 min after administration, the patient developed nausea and dyspnea, necessitating immediate discontinuation of the treatment according to the European Academy of Allergy and Clinical Immunology guidelines for anaphylaxis [6]. Intravenous hydrocortisone (100 mg) was administered twice, after which the symptoms gradually improved. No urticaria, hypotension, or hypoxia were documented.

**Fig. 1:**
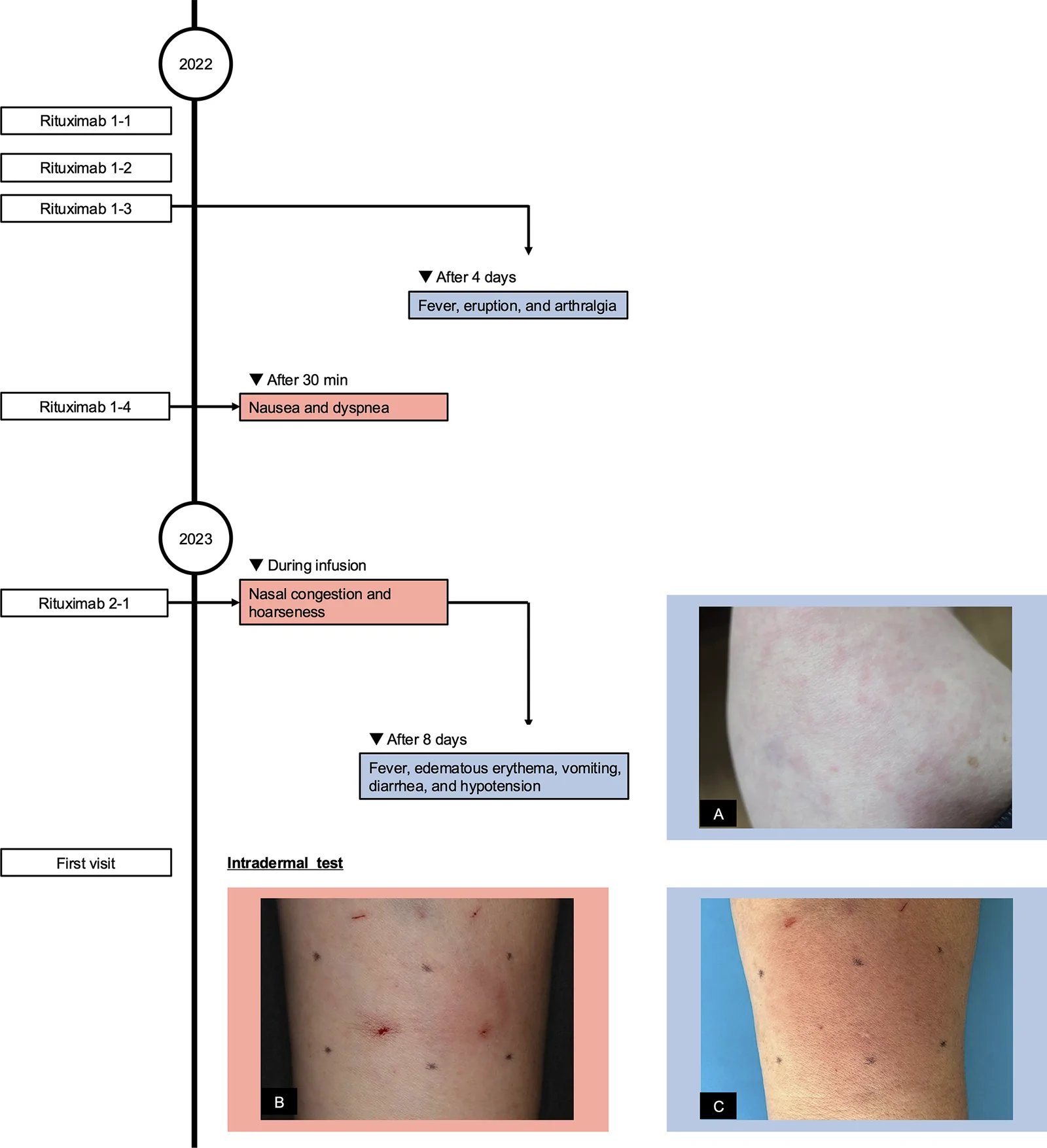
Case report timeline with clinical and intradermal test findings. Rituximab 1–1, 1–2, and 1–3 indicate the first, second, and third administrations in the first course, respectively; 2−1 indicates the first administration in the second course.** A** Edematous erythema on day 8 after rituximab.** B** Wheal on intradermal test day.** C** Worsening erythema the next day

Subsequently, her lymphoma showed slight progression, and 1 year after the initial treatment, the patient began bendamustine–rituximab combination therapy. Bendamustine was administered on days 1 and 2 without adverse events, followed by rituximab infusion on day 6 (375 mg/m^2^), which was administered over a prolonged duration (approximately 3–4 times longer than the standard infusion time) to minimize the risk of severe reactions. Within minutes after the initiation of rituximab infusion, the patient developed symptoms including nasal congestion and hoarseness. Intravenous hydrocortisone (100 mg) was administered, and the patient was able to complete the infusion with resolution of symptoms. Eight days after rituximab infusion (day 14), she presented with fever, vomiting, diarrhea, and hypotension, along with pruritic edematous erythematous plaques on the trunk and extremities (Fig. 1a). Laboratory tests indicated elevated inflammatory markers, including C-reactive protein (19.20 mg/dL) and a leukocyte count of 17,800/µL, with no evidence of bacterial infection, and her symptoms spontaneously improved within 3 days.

HACA levels, measured using an electrochemiluminescence immunoassay (LabCorp, USA), exceeded the assay upper limit (> 5000 ng/mL; reference < 25 ng/mL). Based on the clinical features and markedly elevated HACA levels, the delayed reaction was considered suggestive of RISS; however, HACA levels are not necessarily correlated with RISS. Scratch tests were conducted using bendamustine (2.4 mg/mL), rituximab (2 mg/mL), and polysorbate 80 (0.14 mg/mL), an excipient contained in the rituximab formulation. Serial dilutions from 1:1000 to undiluted (ten-fold increments) were prepared in saline, and all tests yielded negative results. Although skin prick testing is generally preferred over scratch testing, the latter was performed in this case based on institutional practice. Intradermal testing with rituximab (1 mg/mL), a concentration reported to be non-irritant and associated with a low risk of false-positive reactions in a previous study [7], resulted in a positive reaction (Fig. 1b). The wheal initially began to resolve but showed worsening erythema the following day (Fig. 1c), which persisted for at least 2 days before fully subsiding without post-inflammatory pigmentation. A graded challenge test with bendamustine was performed, starting at a 1:100 dilution and increasing ten-fold stepwise up to the undiluted solution. Each step was administered with an observation period of 30 min, and no adverse reactions were observed. Lymphoma treatment was suspended given these results; bendamustine monotherapy was planned if the disease progressed.

## Discussion and conclusions

This case highlights two important findings. First, RISS may follow rituximab-induced anaphylaxis after several days’ delay. Second, skin testing may be a valuable diagnostic tool for identifying immediate-type allergic reactions even in the presence of RISS. In prior cases, anaphylaxis and RISS occurred either during different administrations or overlapped in time during the same infusion (Table 1) [8–11]. To the best of our knowledge, this is the first reported case in which RISS developed several days after the resolution of anaphylaxis triggered by the same rituximab infusion. Our case demonstrated sequential immediate and delayed hypersensitivity reactions following a single rituximab infusion.

**Table 1:** Reported cases of rituximab-induced serum sickness with concurrent rituximab-induced anaphylaxis

	Age	Sex	Background	Rituximab regimen			Time of onset	Clinical presentation	Diagnosis		Diagnostic approach			References
				Dose	Course	Infusion number in course			Anaphylaxis	RISS	Elevated tryptase	HACA	Skin test	
1	86	F	Autoimmune hemolytic anemia	375 mg/m2	2	1	After 5 days	Rash, fever, arthralgia, hypotension	+	+	+	+	NA	[8]
2	6	M	Membranous nephropathy	375 mg/m2	1	1	After 8 days	Arthralgia, myalgia	−	+	NA	NA	NA	[9]
					1	2	After 1 week	Rash, arthralgia	−	+				
					1	3	During infusion	Swelling of lips and periorbital regions, choking sensation, rash, fever	+	-				
3	60	F	Rheumatoid arthritis	1000 mg	1	1	After 6 days	Rash, fever, arthralgia	−	+	NA	NA	NA	[10]
					1	2	Within minutes	Swelling of lips and periorbital regions, choking sensation, tachycardia	+	−				
4	65	F	Sjogren syndrome, MALT lymphoma	375 mg/m2	1	2	After 4 days	Rash, fever, arthralgia	−	+	NA	−	NA	[11]
					1	3	After 30 min	Swelling of the face and lips, dyspnea, nausea, wheezing		−				
					2	2	After 2 days	Fever, arthralgia	−					
Our case	70	F	MALT lymphoma	375 mg/m2	1	3	After 4 days	Rash, fever, arthralgia	−	+	NA	+	+	
					1	4	After 30 min	Nausea, dyspnea	+	−				
					2	1	During infusion	Nasal congestion, hoarseness	+	−				
							After 8 days	Fever, rash, vomiting, diarrhea, hypotension	+༟	+				

RISS, rituximab-induced serum sickness; HACA, human anti-chimeric antibody; MALT, mucosa-associated lymphoid tissue; NA, not applicable

In patients with multiple hypersensitivity reactions to rituximab, careful monitoring after administration and patient education to ensure prompt reporting of delayed reactions are essential. The immediate reaction in this case, occurring at the fourth administration after an uneventful first dose, suggests anaphylaxis as the most likely mechanism. The positive intradermal test observed in our case may support the involvement of an IgE-mediated mechanism. Biphasic anaphylaxis typically occurs within 72 h of the initial symptoms [12]. In the present case, the second reaction occurred 8 days after the initial infusion, suggesting that biphasic anaphylaxis is unlikely and that the reaction may be attributable to RISS. The transition from immediate to delayed reactions may reflect overlapping immunological mechanisms, however, the exact relationship between these processes remains unclear. Previous reports have documented rituximab-induced anaphylaxis occurring 5–11 days after administration, with serum tryptase measurements confirming the involvement of immediate-type hypersensitivity in those cases [8, 13]. In the current case, serum tryptase was not measured at the time of the second reaction, making it difficult to determine whether the delayed response was due to RISS alone or also involved anaphylaxis. In this context, additional diagnostic approaches such as basophil activation testing and characterization of IgE and HACA may be useful to better assess and profile hypersensitivity reactions to rituximab.

The patient’s intradermal reaction exhibited a delayed flare following the initial wheal response (Fig. 1b, c). This sequential pattern parallels the clinical course observed in this case and may suggest a delayed hypersensitivity process. However, delayed local reactions can also occur as IgE-mediated late-phase responses and are not specific for type III hypersensitivity reactions. Type III hypersensitivity reactions are mediated by immune complex formation involving complement-binding IgM or IgG; however, their relationship to positive skin test results has not been fully established [3]. Therefore, the mechanism underlying the delayed reaction in this case remains uncertain. Although its clinical significance warrants further investigation, this case highlights the potential utility of skin testing as a diagnostic tool for rituximab hypersensitivity. It also underscores the importance of monitoring patients for several days after skin testing.

This study has several limitations. Serum tryptase was not measured, which limits the ability to definitively assess the contribution of immediate-type hypersensitivity during the delayed reaction. In addition, total IgG and IgM levels, complement levels (C3, C4), circulating immune complexes, and cryoglobulins were not assessed.

In conclusion, rituximab-induced immediate allergic reactions may coexist with RISS, with delayed symptoms emerging several days after the resolution of initial reactions. Furthermore, in cases wherein multiple hypersensitivity reactions are suspected, skin testing can be useful in profiling these reactions. Establishing standardized diagnostic criteria and elucidating the underlying mechanisms of rituximab-associated hypersensitivity reactions is essential to improving the evaluation and management of RISS.

## Data Availability

The datasets generated and/or analyzed during the current study are available from the corresponding author on reasonable request.

## Abbreviations

RISS: Rituximab-induced serum sickness
MALT: Mucosa-associated lymphoid tissue
HACA: Human anti-chimeric antibodies

## References

[1] Zian Z, Berry SPD, Bahmaie N, Ghotbi D, Kashif A, Madkaikar M, et al. The clinical efficacy of rituximab administration in autoimmunity disorders, primary immunodeficiency diseases, and malignancies. Int Immunopharmacol. 2021;95:107565. 10.1016/j.intimp.2021.107565 33773205

[2] Fouda GE, Bavbek S. Rituximab hypersensitivity: from clinical presentation to management. Front Pharmacol. 2020;11:572863. 10.3389/fphar.2020.572863 PMC7508176 33013416

[3] Karmacharya P, Poudel DR, Pathak R, Donato AA, Ghimire S, Giri S, et al. Rituximab-induced serum sickness: a systematic review. Semin Arthritis Rheum. 2015;45:334–40. 10.1016/j.semarthrit.2015.06.014 26199061

[4] Bayer G, Agier MS, Lioger B, Lepelley M, Zenut M, Lanoue MC, et al. Rituximab-induced serum sickness is more frequent in autoimmune diseases than in hematological malignancies: a French nationwide study. Eur J Intern Med. 2019;67:59–64. 10.1016/j.ejim.2019.06.009 31279430

[5] Lunardon L, Payne AS. Inhibitory human anti-chimeric antibodies to rituximab in a pemphigus patient. J Allergy Clin Immunol. 2012;130:800–3. 10.1016/j.jaci.2012.03.022 PMC3412073 22541247

[6] Muraro A, Worm M, Alviani C, Cardona V, DunnGalvin A, Garvey LH, et al. EAACI guidelines: Anaphylaxis (2021 update). Allergy. 2022;77:357–77. 10.1111/all.15032 34343358

[7] Li L, Wang L, Guan K, Liu J, Zhou D, Zhang Y, et al. Use of skin testing screening and desensitization before the first exposure of rituximab. Cancer Manag Res. 2021;13:9319–28. 10.2147/CMAR.S339282 PMC8710525 34992455

[8] Cottu A, Chaara T, Caré W, Vanquaethem H, Nielly H. Rituximab-induced serum sickness overlapping with anaphylaxis: a case report. Cureus. 2023;15:e38333. 10.7759/cureus.38333 PMC10230849 37266062

[9] Bayram MT, Soylu A, Kavukçu S. Rituximab-induced serum sickness and anaphylaxis in a child with nephrotic syndrome. Turk J Pediatr. 2020;62:884–8. 10.24953/turkjped.2020.05.025 33108096

[10] Kumar A, Khamkar K, Gopal H. Serum sickness and severe angioedema following rituximab therapy in RA. Int J Rheum Dis. 2012;15:e6–7. 10.1111/j.1756-185X.2011.01645.x 22324965

[11] Isoda A, Ishikawa T, Sato N, Miyazawa Y, Matsumoto M, Sawamura M. Repeated rituximab-induced serum sickness with anaphylaxis. Rinsho Ketsueki. 2016;57:771–3. 10.11406/rinketsu.57.771 27384859

[12] Kim TH, Yoon SH, Hong H, Kang HR, Cho SH, Lee SY. Duration of observation for detecting a biphasic reaction in anaphylaxis: a meta-analysis. Int Arch Allergy Immunol. 2019;179:31–6. 10.1159/000496092 30763927

[13] Polito V, Barbacki A, Isabwe G. Type I allergic reaction to rituximab upon first lifetime exposure: a case report. Allergy Asthma Clin Immunol. 2020;16:56. 10.1186/s13223-020-00448-8 PMC7318363 32607108

